# Navigating complex care pathways–healthcare workers’ perspectives on health system barriers for children with tuberculous meningitis in Cape Town, South Africa

**DOI:** 10.1371/journal.pgph.0003518

**Published:** 2024-08-20

**Authors:** Dzunisani Patience Baloyi, Hanlie Myburgh, Danite Bester, Michaile Gizelle Anthony, Juli Switala, H. Simon Schaaf, Lenny Naidoo, Regan Solomons, James Nuttall, Jaco Murray, Ursula Rohlwink, Anthony Figaji, Graeme Hoddinott, Karen Du Preez

**Affiliations:** 1 Department of Paediatrics and Child Health, Desmond Tutu TB Centre, Faculty of Medicine and Health Sciences, Stellenbosch University, Cape Town, South Africa; 2 Amsterdam Institute for Social Science Research (AISSR), University of Amsterdam, Amsterdam, Netherlands; 3 The Aurum Institute, Johannesburg, South Africa; 4 Department of Paediatrics and Child Health, Faculty of Medicine and Health Sciences, Stellenbosch University, Cape Town, South Africa; 5 City Health Department, City of Cape Town, Western Cape, South Africa; 6 Department of Paediatrics and Child Health, University of Cape Town, Cape Town, South Africa; 7 Western Cape Department of Health, Paarl Hospital, Paarl, South Africa; 8 Neuroscience Institute, University of Cape Town, Cape Town, South Africa; 9 Division of Neurosurgery, Department of Surgery, University of Cape Town, Cape Town, South Africa; 10 School of Public Health, Faculty of Medicine and Health, University of Sydney, Camperdown, Australia; University of California San Francisco, UNITED STATES OF AMERICA

## Abstract

Tuberculous meningitis (TBM) occurs when tuberculosis (TB) bacilli disseminate and seed into the meninges, triggering a severe inflammatory response that often leads to brain infarction. It is the most severe and debilitating form of childhood TB with high mortality, and children who survive TBM often suffer lifelong physical and neuro-disability resulting in emotional, social, and economic burdens for families. In the early stages the symptoms may be non-specific and so the diagnosis is often made late when the patient already has significant brain injury. To facilitate earlier diagnosis, it is important to understand how patients are evaluated. This study aimed to chart health systems for paediatric TBM care at both primary healthcare (PHC) and hospital level in Cape Town, South Africa. We conducted fourteen in-depth interviews and eight days of semi-structured observations of patient flow across eight healthcare facilities. We found that children with TBM navigate multiple levels of care categorised into pre-admission and primary care, hospital admission and inpatient care, and post-discharge follow-up care. Healthcare workers identified the following health system barriers along the TBM care pathway for children: limited post-training and mentorship opportunities to manage TBM, overburdened facilities, time constraints, lack of recognition of TBM symptoms, delays in referral between PHC and hospital, lack of standardized diagnostic algorithms, limited diagnostic tests and a lack of child-friendly, easy-to-administer treatment. Regular and compulsory training on TB and TBM in children, including continuous mentoring and support to healthcare workers working in child health and TB services in high TB-burden settings, can facilitate early recognition of symptoms and rapid referral for diagnosis. Algorithms outlining referral criteria for patients with possible TBM at both PHC facilities and district level hospitals can guide healthcare providers and facilitate timely referral between different levels of healthcare services. An integrated data system and alert functions could flag multiple healthcare visits and improve communication between different healthcare facilities during diagnosis and treatment. Children and families affected by TBM are an especially vulnerable sub-population requiring high priority attention and support.

## Introduction

Tuberculosis (TB) remains one of the top ten causes of mortality among children younger than five years. It affected an estimated 1,25 million children (<15 years of age) in 2022, with 214,000 children dying of TB in that year [1]. TB meningitis (TBM) occurs when *Mycobacterium tuberculosis* bacilli disseminate and seed to the meninges, triggering a severe inflammatory response [2]. TBM is the most severe and debilitating form of childhood TB with high mortality and morbidity, children who survive TBM often suffer lifelong neuro-disability resulting in emotional, social, and economic burdens for patients and their families [3]. A systematic review and meta-analysis including data from 19 studies and 1636 children found that children presenting with British Medical Research Council (BMRC) stage 3 TBM–the most advanced clinical stage–were significantly more likely to die during treatment or to develop neurological sequelae [4]. This is likely due to the development of secondary complications of TBM by the time diagnosis is made, the most important of which are vasculitis (leading to stroke) and hydrocephalus. These are consequences of the exaggerated host inflammatory response that are difficult to manage and are better avoided by the early initiation of therapy. A deterioration in the level of consciousness and the development of neurological deficits are indicators of cerebral impairment that may or may not be reversible [3]. Early diagnosis, referral, treatment initiation and rehabilitative services are therefore imperative to optimize treatment outcomes and reduce TBM-related mortality and morbidity.

Diagnosing TBM is challenging as children often present with non-specific symptoms, especially in the early stage of disease [4]. Currently there is no highly sensitive or specific point-of-care diagnostic test for TBM which means that diagnosis mostly occurs at tertiary level hospitals where critical care and advanced imaging are available. Timely diagnosis of TBM is thus dependent on caregivers’ recognition of and healthcare-seeking response to early symptoms, as well as a high index of suspicion and rapid referral by healthcare workers (HCWs) at primary healthcare (PHC) level. Treatment and supportive care for children with TBM can require prolonged hospitalisation; where adequate resources and support are available; however, outpatient treatment can be successful [5]. Post-treatment, multidisciplinary care is the ideal approach to address the child’s needs that depend on age and degree of neurological sequelae [6]. Children with TBM thus navigate multiple levels of the healthcare system before, during and after their treatment journey, requiring well-functioning underlying health systems.

There has been on-going clinical research on TBM in the Western Cape province, South Africa, since 1985 [7], including diagnostic, neurophysiological, treatment and operational research studies [8–11]. TBM has been reported as the most common form of bacterial meningitis at one of the tertiary referral hospitals in the Western Cape between 2007–2009 (126/557, 22%) [12]. Ongoing TBM surveillance at this hospital reported an alarming increase in the number of children admitted with TBM in the paediatric neurology service in 2017 (incidence rate ratio = 2.2, p<0.001; 2017 = 70 vs. mean 2014–2016 = 32.7), following global Bacillus Calmette-Guérin (BCG) vaccine shortages [13]. Another tertiary referral hospital reported that 90% (40/44) of patients presented with Stage II or III TBM, with a mortality rate of 16% (7/44) [14]. Neurological disability amongst children who survived ranged from persistent vegetative state (n = 3/37; 8.1%) and severely disabled (n = 2/37; 5.4%), to mild and moderate disability (n = 11/37; 29.7%) [14].

As an initial step to identify health system gaps and interventions, we charted paediatric TBM care pathways at hospital and primary healthcare levels in Cape Town, South Africa. HCWs’ perspectives were included to understand the impact of COVID-19 on patient pathways and the health system, and to highlight areas for improvement.

## Methods

### Study design

Formative research included qualitative and observational data collected in preparation for establishing a prospective TBM cohort study.

### Setting

The Western Cape province comprises one metropolitan municipality known as City of Cape Town (COCT). Within this area, there are more than 100 PHC facilities, including mobile and satellite facilities. The COCT is divided into eight sub-districts. Two tertiary referral hospitals serve the entire paediatric population in the province, i.e., Tygerberg Hospital, and Red Cross War Memorial Children’s Hospital. Brooklyn Chest Hospital is a specialised TB hospital where the majority of paediatric TB patients requiring prolonged hospitalization are admitted.

### Sampling

We purposively sampled six PHC facilities from two sub-districts in Cape Town and included two tertiary hospitals, one specialized TB hospital and one district hospital to be qualitatively representative of the health system in Cape Town. PHC facilities were conveniently sampled from two sub-districts with the support from the district managers, based on paediatric TB burden and trends, using TB data from 2019 and 2020. We worked with these two sub-districts due to limited resources and feasibility. We consulted with Department of Health stakeholders to select facilities using routine TB data. We reviewed the following performance indicators; percentage children of the total TB case load for 2019 and 2020; ratio of 0–4 vs 5–14-year-olds; change in adult TB case load– 2020 vs 2019; change in paediatric TB case load– 2020 vs 2019; change in percentage of children 2020 vs 2019 (i.e., relative change in paediatric TB compared to adult TB caseload). At each health facility we purposively sampled 2 to 3 HCWs for diversity in role (e.g., nurse, doctors, and specialized medical staff) and for ‘rich cases’.

This is a purposive sampling technique where participants are selected because they have in-depth or expert knowledge with a particular program or patient group for example [15]. An example would be inviting nurses with multiple years of experience delivering a particular service to participate in a study, rather than including any nurse involved in delivering that service in a sample. The sampling logic is about maximizing collection of data with participants with extensive experience to make good use of limited spots in a ‘small’ qualitative sample.

### Data collection

We collected data between 1 November 2021 and 28 February 2022, conducting in-depth interviews with nurses and doctors (n = 14), semi-structured observations (one day of observations per facility), including informal conversations with staff to help understand patient flow. We used a study-specific discussion guide to facilitate interviews, which included questions on the existing TBM care pathway, barriers that impact patients’ progression along the care pathway, and the impact of COVID-19 on access to health services. Interviews were conducted in participants’ preferred language–English or Afrikaans–and lasted approximately 30–45 minutes.

### Analysis

We summarised the interview and observation data into case descriptions of the TBM care pathway at each facility. We followed an iterative process to inductively analyse these case descriptions in a Microsoft Excel Spreadsheet, to describe the health systems for the paediatric TBM care pathway at hospitals and at PHC facilities, as well as barriers identified across the TBM care continuum.

### Ethics considerations

We obtained ethics approval for the study from the Health Research Ethics Committee at Stellenbosch University (N21/04/041), and approval for the study to be conducted from the Western Cape Provincial Department of Health (WC_202107_021) and Cape Town City Health (Project ID: 9434). All participants voluntarily consented to be interviewed and completed written informed consent.

### Findings

**Fig 1:**
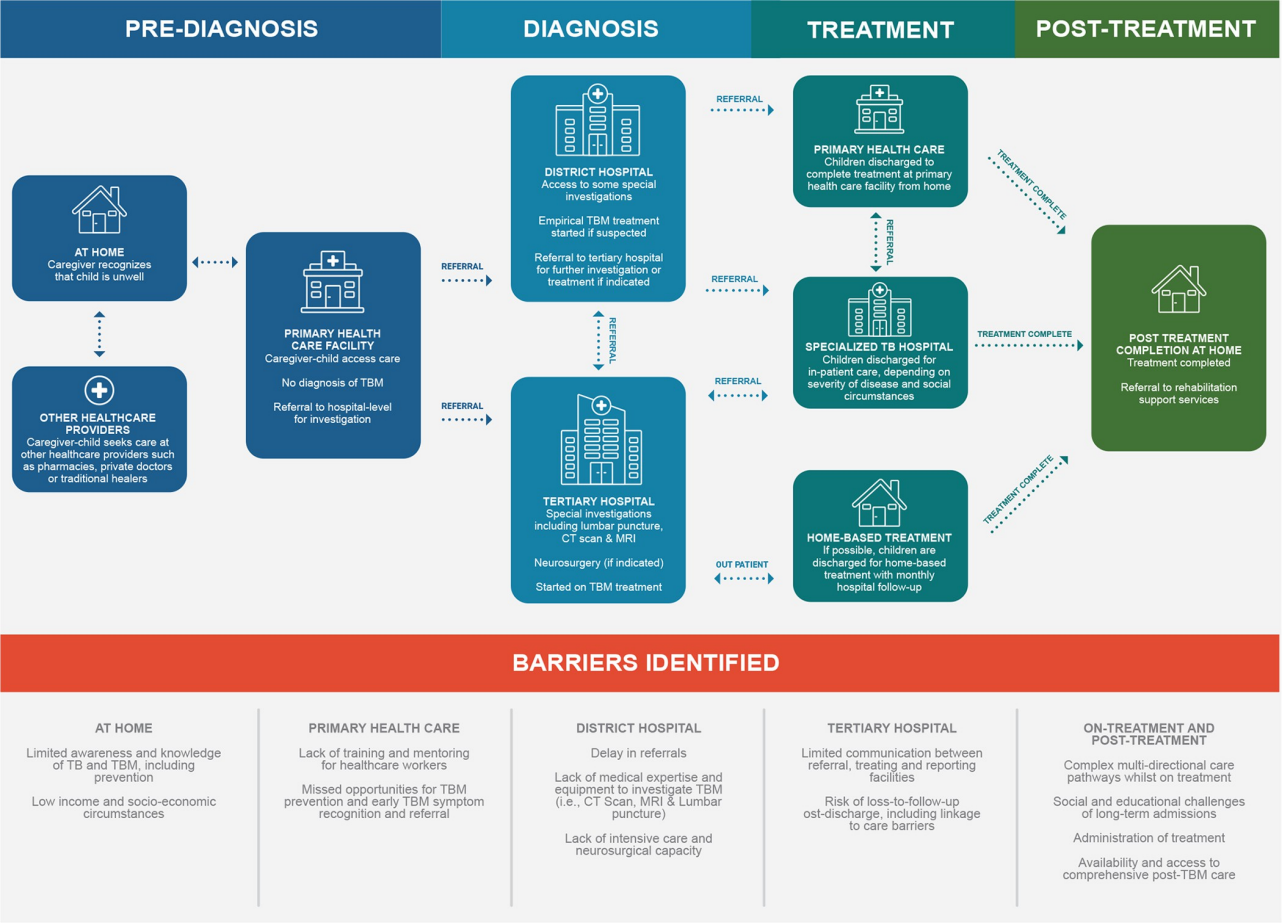
Paediatric tuberculous meningitis care pathways and barriers identified by healthcare workers (HCWs). Children with probable TBM enter care mainly through walk-in into Primary Health Care (PHC). Grey arrows indicate movement between various levels of care. Both-sided arrows indicate that children could have more than one encounter at the facility and move back and forth between facilities. After first encounter at PHC, children require a referral from PHC to district, tertiary and specialized TB hospitals. Some children are discharged from tertiary hospital for home-based care treatment. The bottom of the page indicates several identified barriers that hinder and prevent children from progressing into appropriate care. CT = computed tomography; MRI = magnetic resonance imaging; TBM = tuberculous meningitis; TB = Tuberculosis.

We charted the health systems for paediatric TBM care at both PHC and hospital level in Cape Town, South Africa and present HCWs’ perspectives on barriers that impact patients’ progression along this care pathway. Fig 1 provides an overview of the existing care pathways as well as barriers identified at each step. We also explored HCWs’ suggestions on optimizing the TBM care pathway.

### Overview of paediatric TBM care pathways

We divided the pathway into pre-admission and primary care, hospital admission and inpatient care, and post-discharge follow-up care.

### Pre-admission and primary care

This pathway begins with caregivers recognizing symptoms and seeking care at a PHC facility, the initial point of contact with HCWs. Most PHC facilities in COCT are nurse-run with doctor support from the larger facilities. After receiving a patient folder, the child is referred to the weighing room for observations and then to child-health services for evaluation of symptoms of pulmonary TB. Nurses screen for TB and manage symptoms based on Integrated Management of Childhood Illness (IMCI) guidelines [16]. Children presenting with early non-specific TBM (or other extrapulmonary TB) symptoms like appetite loss, reduced playfulness, and vomiting are often treated symptomatically, resulting in numerous visits before referral or correct diagnosis. However, those with more severe symptoms including seizures, decreased consciousness, and high fever are rapidly triaged for emergency assessment by a nurse or doctor, if available, to determine the need for urgent hospital referral. Thereafter, caregiver-child dyads will be referred to the next level healthcare facility by ambulance. HCWs emphasized that definitive diagnosis rarely occurs at the PHC level given limited resources for lumbar puncture, computed tomography (CT) scans, and other essential investigations. Thus, most possible TBM cases at PHC level are referred to district, regional or provincial hospitals based on proximity and symptom severity.

### Hospital admission and inpatient care

Most children enter hospital admission and inpatient care through a referral mechanism pathway. However, some children do access care without a referral from PHC, especially those with advanced TBM symptoms such as seizures or decreased level of consciousness. Children who are severely ill or unstable are transferred through inter-facility transport. Those considered not seriously ill but referred from PHC to hospital for further investigations must use private or public transport. These next level of care facilities have specialised medical staff (paediatric doctors and specialists) as well as neuroimaging necessary to make a diagnosis of TBM. HCWs will start by stabilizing children according to their presenting symptoms, assess vital signs and conduct basic clinical examination, laboratory and radiological investigations including chest radiography and, if safe, do a lumbar puncture. At this point, prior to any microbiological confirmation, children are often treated empirically for both bacterial meningitis and TBM. Based on the results of investigations, children are usually referred for neuroimaging to support the diagnosis and establish the need for additional interventions such as neurosurgery. At the tertiary healthcare level, HCWs reflected that they receive referrals from both the PHC and district level to conduct additional diagnostic investigations and consult with sub-specialists. Children with hydrocephalus will be investigated by paediatric neurologists and/or neurosurgeons and referred for neurosurgical intervention if indicated. Thereafter children will be managed in a paediatric ward or intensive care unit. HCWs reflected that children are hospitalised until they are stable on TBM treatment and will be discharged to continue treatment at home or as an in-patient at a specialised TB hospital, depending on the level of available support at home and the child’s specific treatment needs.

### Post-discharge follow-up care

At the tertiary level of care caregiver-child dyads will be evaluated by a multi-disciplinary team prior to discharge to determine their suitability to enter a home-based care programme [5, 17, 18]. The team typically includes a paediatrician (or paediatric neurologist), social worker, occupational therapist, and physiotherapist. If children require additional supportive care during treatment, often due to severe impairment or disability, or if they do not have adequate social support to receive home-based care, they will be transferred to a TB hospital for in-patient hospital treatment [11]. The duration of hospitalization depends on multiple factors, but the child’s health takes priority. HCWs described that children continuing treatment on the home-based care programme are followed up monthly at the tertiary hospital.

### Barriers identified across the TBM care continuum

We categorized identified barriers into pre-admission and primary care, hospital admission and inpatient care, and post-discharge follow-up care but also included barriers associated with COVID-19 restrictions.

### Pre-admission and primary care barriers

Barriers prior to accessing care at PHC level included delayed recognition of TBM symptoms by caregivers and poor socioeconomic circumstances. Limited post-training mentorship to recognize the early symptoms of TBM at PHC level, overburdened facilities and time constraints were identified as barriers at PHC level.

Most HCWs reflected and recognized that PHC facilities often missed children presenting with early TBM symptoms, even when there was a history of TB exposure. HCWs attributed these missed opportunities to recognize TBM symptoms to a lack of post-training mentorship and limited exposure to diagnosing TBM in children, which mostly occurred in hospital. However, these facilities were the first point of care for children with probable TBM symptoms, and early recognition and referral are critical. A doctor from a tertiary hospital explained:

*“Looking back at some of the families caring for children diagnosed with TBM*, *they reported going to a clinic [PHC]*. *Their children were misdiagnosed with teething*, *flu*, *or gastro*. *TBM is very hard to detect especially at PHC level*. *They will only consider TBM if the child presents with a seizure*.*”*

HCWs recognised that PHC facilities were overburdened and were constrained to see all patients seeking care on a given day. This led HCWs to rush through consultations and to take incomplete patient histories, which could help to identify possible TB exposure; children presenting with potential TBM symptoms were not always examined thoroughly. A district manager reflected:

*“They [healthcare workers] are trained to identify these [TBM] symptoms*, *they experience challenges when clinics are full of patients and screening just becomes completing a tick box*. *Healthcare workers are not attentive to the danger or risks signs of disease*, *for instance TB contact in the household of a sick child*.*”*

HCWs described that caregivers often struggled to recognize that their children were unwell, due to the non-specific nature of TBM symptoms, which delayed seeking health care. For instance, a HCW emphasized:

*“Caregivers cannot pick up the growth faltering between ages two- to six-year old*, *until the child presents with severe symptoms such as seizures and acutely ill at the hospital*.*”*

*“A child with flu-like symptoms*, *parents (are) less likely to seek help*. *However*, *for the extreme cases where the child with possible TBM cannot move*, *parents do seek help*.*”*

HCWs explained that patients’ challenging socio-economic circumstances often prevented them seeking care at PHC facilities especially if they needed to travel out of their communities. Many communities in Cape Town experience regular periods of gang violence, community unrest and transport strikes which at times made accessing PHCs unsafe and impossible. A nurse explained further:

*“Most patients come from poor circumstances*, *and they want their children to be healthy and well*. *They often struggle with money to come to the facility for follow-ups*.*”*

### Hospital admission barriers

Barriers to care at district and tertiary levels included delay in referral pathways between PHC and hospitals, and limited sensitive and specific diagnostic tests for TBM.

HCWs described that further investigations at hospital level were often delayed due to poor communication between health worker cadres. A HCW reflected:

*“There are often difficulties between healthcare workers during the referral process*. *For instance*, *doctors at secondary level will question the nurses at PHC when they refer patients*. *In such cases it delays patients receiving care*.*”*

HCWs recognized that a diagnosis of TBM requires several investigations due to the lack of sensitive diagnostic tests. HCWs emphasized:

*“The diagnostic tools that diagnose childhood TBM have low sensitivity*. *We require better diagnostic tools due to low sensitivity of the existing tools*. *The diagnosis for TBM is based on clinical features*, *scans*, *and tests*. *It is challenging to identify TB in brain fluid*.*”*

### Post-admission and post-discharge barriers

Post-admission and post-discharge (for treatment) barriers include lack of palatable and easy-to-administer medication, as well as limited post-TBM rehabilitation services and poor socio-economic circumstances of the families. HCWs reflected that current TBM treatment is not palatable for children, making it difficult to administer to children especially when they are discharged to continue treatment from home. For instance, a HCWs described:

*“If we [healthcare workers] had palatable drugs*, *this would make our jobs easier*. *For instance*, *a prescribed drug [ethionamide] has an ugly taste*, *children usually spit it out and it makes the children feel nauseas*.*”*

*“They [caregivers] struggle to break up tablets as there are no child-friendly medication*. *Some caregivers prefer their children to take their treatment from home*.*”*

In Cape Town, some children are not considered for post-TBM rehabilitation services, partly because access to and availability of these services are very limited. The availability of neurodevelopmental, psychological or educational assessments are also very limited in South Africa’s public health sector, as is the availability of special schooling for children with special needs, including children suffering with post-TBM disability or behaviour challenges.

### COVID-19 related barriers

Data collection for this study coincided with the COVID-19 pandemic and response, and HCWs shared how the pandemic compounded existing health systems challenges for the prevention, diagnosis, treatment, and rehabilitation of children with TBM. HCWs identified barriers that included limited access to PHC services due to COVID-19 related lock down and restrictions, missed opportunities for prevention (i.e., TPT and BCG) and to screen for and diagnose TB and TB-related disease (including TBM), and reduced post-hospitalization follow-up and care options.

During data collection, some PHC facilities implemented an electronic appointment system whereby patients could access care through a scheduled appointment only and this system was adapted to reduce transmission of the COVID-19 virus. This service adaptation possibly increased delays in diagnosis and referral of children presenting with probable TBM symptoms. HCWs reflected:

*“We (facility) did not do any walk-in’s*, *and patients would have to go to the day hospital to be assisted*. *Patients were given appointment dates to come to the facility*, *however if they do not show up*, *they return for another appointment date*.*”*

HCWs highlighted that there was more emphasis on the screening and testing of COVID-19 symptoms. Other HCWs reflected that they struggled to recognise the difference in COVID-19 and TB symptoms, resulting in missed opportunities of diagnosing TB disease. HCWs reflected:

*“COVID-19 pandemic had an impact on the diagnosing of TB*, *due to the similarities of the symptoms*. *There was more emphasis on testing for COVID-19 as opposed to TB in general*.*”*

HCWs reported that prior to the COVID-19 pandemic uptake on TPT was sub-optimal, with many eligible candidates not initiated. For those who were initiated on TPT, completion rates remained poor. HCWs believed these numbers were underreported during COVID-19as fewer patients sought care at the facilities and fewer were diagnosed with TB during this time. HCWs described:

*“There were less patients seeking health care at facilities*, *and less patients diagnosed with TB*. *This implied that more children were not initiated on TPT*.*”*

*“There is still low coverage of TPT*. *About 70% of children that are initiated*, *do not complete TPT*.*”*

HCWs highlighted that BCG vaccinations were available in facilities; however, they still encountered children who were unvaccinated. From a logistics perspective facilities usually batched children in groups before opening a BCG vial to maximise use and avoid wasting. However, this process also posed challenges for caregivers and children. For instance, a HCW shared:

*“Caregivers can go to the facility for catch-up vaccinations and they might not have stock or an opened BCG vial on the day of return appointment*.*”*

HCWs explained that efforts and resources were redirected towards the COVID-19 pandemic. For instance, a TB hospital was converted to a COVID-19 specialising hospital which affected the TBM follow-up pathway. One HCWs shared:

*“There was a massive impact on follow-up processes*. *Prior to COVID*, *children diagnosed with TBM were managed at a local TB hospital*. *However*, *during the COVID-19 pandemic the one TB hospital was changed to a COVID hospital and closed for TB services*. *Patients were discharged to another hospital further away from home and other patients were treated through a home-based care programme*.*”*

## Discussion

These are the first qualitative and observational data describing the health systems for paediatric TBM care in a high TB-burden setting. We also identified barriers that prevent children from progressing along the TBM Care pathway.

Most children with TBM will navigate across multiple levels of health services for diagnosis and treatment, including PHC, district hospitals, tertiary hospitals (centrally located) and specialized TB hospitals. Accessing these health services does not follow a linear pathway (Fig 1) but is often complex and particular to the severity of a child’s symptoms at presentation and the accessibility of services in the community. These complex pathways likely increase the risk of disease progression and worsen outcomes of children. Good communication and strong referral pathways between different levels of care is therefore an essential part of the TBM Care pathway.

Consistent with our findings, barriers such as the delayed recognition of symptoms, lack of post-training mentorship at PHC level, delayed referrals from PHC to hospital and the complexity of diagnosis of TBM have also been noted in other studies [1, 16, 19, 20]. One of these studies evaluated 30 children diagnosed with TBM and found that 21 (70%) reported TB exposure and the median number of health care visits before diagnosis was 4.0 (range 1–6) [16]. Early diagnosis of TBM relies on a high index of suspicion and careful history taking by front-line HCWs. In the early stages, TBM is often recognised due to a pattern over time rather than at one particular visit. As such, plotting weight and using the “Road-to-Health Booklet (RTHB)” (a health-tracking booklet issued to all children in South Africa upon birth) to document seemingly minor ailments can assist with earlier pattern recognition. Although this is non-specific, it is a common symptom and should be a red flag, especially in the presence of known TB exposure. Given the non-specific nature of TBM symptoms in the early stages of disease, there is a real need for a bedside test either for TB diagnosis or as a biomarker of brain involvement to raise suspicion of TBM.

In our study, HCWs at all levels of care agreed that a heightened suspicion of TBM is needed to facilitate earlier diagnosis. One possible solution to facilitate this is to lower current thresholds for lumbar puncture and brain imaging in children with possible TBM. However, such procedures are not without risk for paediatric patients and will impact on service demands. Clear guidance on referral criteria provided at lower levels of care could help front-line HCWs to timeously identify and refer children with possible TBM. At the same time, clear guidance on the criteria for special investigations should be provided at higher levels of care. Finding the right balance between increasing sensitivity (finding more cases earlier) and reducing specificity (unnecessary investigations [lumbar punctures and CT scans] in children without TBM) is difficult, especially in settings where healthcare systems are already strained and overburdened. Research to document the impact of lowering the threshold for special investigations on the benefit and risks to patients and the healthcare services to determine optimal referral thresholds is needed to inform guidelines.

**Fig 2:**
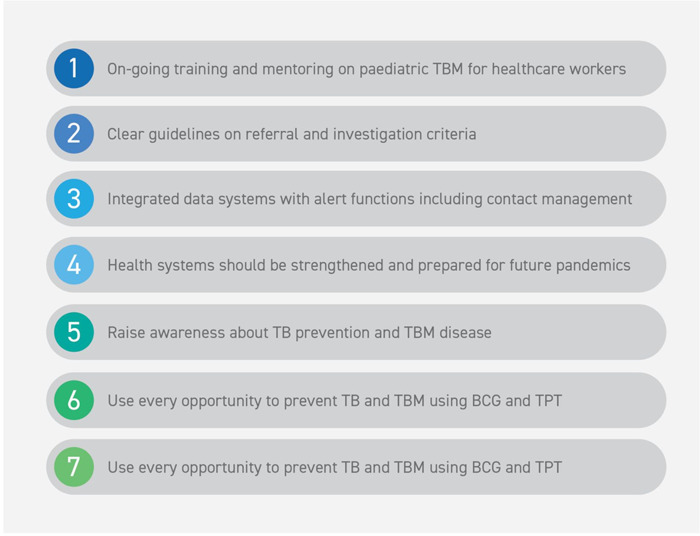
Recommendations to strengthen the TBM care pathway for children in South Africa. TBM = Tuberculous meningitis, TB = Tuberculosis, BCG = Bacillus Calmette-Guérin; TPT = TB preventive therapy.

Based on our study findings, we have outlined recommendations which could improve TBM care pathways for children in South Africa, and possible other high TB-burden settings (Fig 2). HCWs across all levels of care identified a clear need for on-going training and mentoring for diagnosis and management of children with TBM. Clear guidelines on referral and investigation criteria can improve communication and speed up referral processes between facilities. Improved documentation of TB contact management and integrated data systems with alert functions can support HCWs to identify possible TB exposure in children and also facilitate retention in care as children move along the TBM Care pathway. Health systems should be strengthened and prepared for future pandemic, so we can continue delivering health care for other conditions such as TB during a pandemic. Community health education campaigns can raise awareness and influence health seeking behavior amongst caregivers, specifically on TB prevention and early recognition of TB and TBM symptoms in children. For instance, provide pamphlets with information about TBM symptoms and disease presentation in the preferred local language. This information is still limited, especially for the community. Every opportunity we have to prevent TB and TBM should be prioritized. BCG vaccination at birth provides partial but important protection against TBM [13, 21] and TB preventive therapy should be provided to all children following TB exposure to prevent TB and TBM [22]. For example, we could have a health promotion drive to promote BCG and TPT in the communities. These prevention strategies are available in our facilities; however, they are still underutilized.

Collaborative and multi-disciplinary support from healthcare workers is needed to address post-TBM needs comprehensively.

This study has several strengths and limitations. It provides valuable insight into the paediatric TBM care pathway and barriers along the pathway which can be used to inform ideas to change systems to improve clinical care. The research however did not measure the impact of changes on patient outcomes or what the impact will be on health services. Important work for the future would be to understand how many patients could have been investigated earlier if we lowered the threshold for suspicion and investigations, as well as how many patients investigated for, or treated for TBM, did in fact not have the disease. Research to provide clear referral and investigation criteria can help us understand the trade-offs and evaluate the clinical impact as well as the health system impact. Our study was focused only on pathways within the City of Cape Town, and as care pathways could be different depending on the setting, this potentially limits the generalizability of our findings to other settings. Furthermore, our HCW sample was relatively small and the PHC facilities included was a convenience sample from one sub-district. However, HCWs working at hospital-level experience referrals from PHC facilities across the district and their experiences were therefore not limited to one sub-district. Formally reviewing post-TBM health systems were not within the scope of this project. Given the morbidity of TBM, future research should also explore the post-TBM impact on the child and their family and cost to the health system as well as the availability of post-TBM health services in low -and middle—income countries [23].

## Conclusion

Children and families affected by TBM are a vulnerable sub-population requiring high priority attention and support. Even though a small proportion of children with TB develop TBM, the impact of a delayed TBM diagnosis is devastating and can have lifelong implications for the child, family, and health services. Further research should include patient perspectives to fully understand barriers and inform interventions which can strengthen health systems for children with TBM.
